# Raman Spectroscopy Enables Non-invasive and Confirmatory Diagnostics of Salinity Stresses, Nitrogen, Phosphorus, and Potassium Deficiencies in Rice

**DOI:** 10.3389/fpls.2020.573321

**Published:** 2020-10-22

**Authors:** Lee Sanchez, Alexei Ermolenkov, Sudip Biswas, Endang M. Septiningsih, Dmitry Kurouski

**Affiliations:** ^1^Department of Biochemistry and Biophysics, Texas A&M University, College Station, TX, United States; ^2^Department of Soil and Crop Sciences, Texas A&M University, College Station, TX, United States; ^3^The Institute for Quantum Science and Engineering, Texas A&M University, College Station, TX, United States

**Keywords:** nutrient deficiency, rice, Raman spectroscopy, salinity stress, non-invasive diagnostics

## Abstract

Proper management of nutrients in agricultural systems is critically important for maximizing crop yields while simultaneously minimizing the health and environmental impacts of pollution from fertilizers. These goals can be achieved by timely confirmatory diagnostics of nutrient deficiencies in plants, which enable precise administration of fertilizers and other supplementation in fields. Traditionally, nutrient diagnostics are performed by wet-laboratory analyses, which are both time- and labor-consuming. Unmanned aerial vehicle (UAV) and satellite imaging have offered a non-invasive alternative. However, these imaging approaches do not have sufficient specificity, and they are only capable of detecting symptomatic stages of nutrient deficiencies. Raman spectroscopy (RS) is a non-invasive and non-destructive technique that can be used for confirmatory detection and identification of both biotic and abiotic stresses on plants. Herein, we show the use of a hand-held Raman spectrometer for highly accurate pre-symptomatic diagnostics of nitrogen, phosphorus, and potassium deficiencies in rice (*Oryza sativa*). Moreover, we demonstrate that RS can also be used for pre symptomatic diagnostics of medium and high salinity stresses. A Raman-based analysis is fast (1 s required for spectral acquisition), portable (measurements can be taken directly in the field), and label-free (no chemicals are needed). These advantages will allow RS to transform agricultural practices, enabling precision agriculture in the near future.

## Highlights

We show that Raman spectroscopy can be used for pre-symptomatic diagnostics of nutrient deficiencies in rice caused by a lack of nitrogen, phosphorus, and potassium. We also demonstrate that Raman spectroscopy is capable of detecting salinity stresses.

## Introduction

Plants experience a wide range of environmental stresses that inhibit growth and reduce their ability to carry out normal cellular functions (Farber et al., 2019a). These stresses can have abiotic and biotic origins. Biotic stresses can be caused by various pathogens, including bacteria, viruses, and fungi. These pests significantly affect the maturation of crops, reducing their productivity, and they can ultimately destroy entire agricultural ecosystems (Food and Agriculture Organization of the United Nations, 2009). Significant losses of crop yield can also be caused by different abiotic stresses such as salinity, drought, and nutrient deficiency (Pandey et al., 2017). Soil salinity is a global problem, especially in numerous developing countries. High osmotic pressure under salinity stress in the soil prevents water and mineral uptake by plants, which drastically reduces crop yields and, ultimately, the productivity in the high salinity areas. Nitrogen (N) deficiency results in impaired chlorophyll biosynthesis, which leads to poor plant growth and leaf chlorosis (Ding et al., 2005). Potassium (K) and phosphorus (P) deficiencies also cause reduced plant growth as well as brown tips on leaves. Timely detection and identification of these nutrient deficiencies can be used for a site- and dose-specific administration of fertilizers that will mitigate losses associated with these deficiencies (Waraich et al., 2012).

Despite the benefits, confirmatory identification of nutrient deficiencies and salinity stress is a challenging task. Currently, many chromatographic and colorimetric procedures are available for nutrient analysis in both plants and soil. For example, total nitrogen can be determined by nitrate extraction from plant samples using a 1 M KCl solution (Sáez-Plaza et al., 2013). Following nitrate reduction to nitrite using a cadmium column, the concentration of nitrites can then be determined by spectrophotometric measurement (Sáez-Plaza et al., 2013). High temperature combustion, atomic absorption spectroscopy, and atomic absorption spectrophotometry (ICP) offer more advanced approaches for plant nutrient analyses. However, all these methods are destructive, as well as time- and labor-consuming. They also require samples be shipped to analytical laboratories and the use of dangerous chemicals, which makes these analyses expensive and toxic.

Unlike nutrient deficiencies, soil salinity can only be determined by chemical analysis of the soil. Salinity is most commonly determined by measuring the conductivity of soil in water or by using electromagnetic soil sensors (Zaman et al., 2019). A non-invasive alternative to these analytical procedures is strongly desired.

Imaging methods, including thermography, hyperspectral, and RGB, can be used to diagnose plant stresses by detecting changes in the color, texture, or temperature of the plant. If measured from a plane or UAV, these imaging methods enable the monitoring of large agricultural territories (Baena et al., 2017). However, they have not achieved broad application in agriculture due to their poor specificity, complex data analysis, and long image processing times.

Raman spectroscopy (RS) is a non-invasive and non-destructive technique that can be used to probe the structure of samples (Farber et al., 2019a). It is based on inelastic light scattering by molecules that are being excited to higher vibrational or rotational states. Our group has developed techniques to use RS for confirmatory diagnostics of fungal diseases on corn, wheat, and sorghum (Egging et al., 2018; Farber and Kurouski, 2018). We also showed that RS could be used to detect viral diseases of wheat and rose, as well as the presence of bacteria that cause Huanglongbing (HLB or citrus greening) on citrus trees (Farber et al., 2019b; Sanchez et al., 2019a,b). This diagnostic approach is based on the detection of pathogen-induced changes in the structure and composition of plant molecules. Such changes are unique for each pathogenic species. Thus, RS has species-level sensitivity in pathogen diagnostics.

This work evaluates whether abiotic stresses can be detected and identified using RS. We grew rice (*Oryza sativa*) in hydroponic conditions with induced N, P, and K deficiencies as well as low and high salinity stresses. Using a hand-held Raman spectrometer, we collected spectra from the leaves of rice plants before and after inducing these abiotic stresses. In parallel, we made height and chlorophyll measurements, which are often performed in both plant biology and plant breeding to determine progress in plant vegetation and detect possible nutrient deficiencies.

## Materials and Methods

### Plant Materials and Set Up

Presidio, a high yielding rice variety with good grain quality, was used for this study (Wilson, 2009). Pre-germinated rice seeds were transplanted into circular cutouts made in Styrofoam lined with mesh, according to the previously described method (Razzaque et al., 2017). For the first 24 h, these seeds were placed in distilled water. Afterward, Yoshida solution (solution composition is described in the SI) (Yoshida et al., 1976) was used for all six groups, and then the germinating seeds were grown to an age/size suitable for the experiment. Two replications with 30 seeds per replication were used for the study. After 11 days, the seedlings were maintained in Yoshida solution with all macro and micro nutrients for the control group, while seedlings in each of the stress groups {Nitrogen deficient (ND), phosphorus deficient (PD), potassium deficient (KD), medium salt stress [80 mM NaCl (80 mM)], and high salt stress [120 mM NaCl (120 mM)]} were placed in their respective stressor solutions at pH 5.0, which was adjusted daily. Spectral acquisitions, as well as height and chlorophyll measurements, were taken at days 2 (D2), 4 (D4), 6 (D6), 8 (D8), 11 (D11), and 13 (D13) after introduction to stress. Growth and measurements were completed in a growth chamber that maintained relative humidity of 55% under 12 h/12 h (day/night) and temperature at 29°C/26°C (day/night).

### Raman Spectroscopy

Raman spectra were collected with a hand-held Resolve Agilent spectrometer equipped with an 830-nm laser source. The following experimental parameters were used for all collected spectra: 1 s acquisition time, 495 mW power, and baseline spectral subtraction by device software. Previously reported experimental results demonstrated absence of photodegradation of plant material at these experimental conditions (Sanchez et al., 2019a). We also observed neither visual signs of laser-induced photodegradation of rice leaves during spectral acquisition nor any noticeable structural changes in plants in the control group of plants (Supplementary Figure S1). Fifty spectra were collected from each group of plants. Spectra shown in the manuscript are raw baseline corrected, without smoothing.

### Multivariate Data Analysis

PLS_Toolbox (Eigenvector Research Inc.) was used for statistical analyses of the collected Raman spectra. All imported spectra were scaled to unit variance to give all spectral regions equal importance. The first derivative was taken from Raman spectra with a filter width of 45 and polynomial order 2; spectra were median centered. Partial least squares discriminant analysis (PLS-DA) was performed to determine the number of significant components and identify spectral regions that best explained separation between the classes. Analyzed spectra, containing wavenumbers 350–2000 cm^–1^, were used to build PLS-DA models that are discussed in the manuscript.

## Results and Discussion

### Raman-Based Diagnostics of Nutrient Deficiencies

**FIGURE 1:**
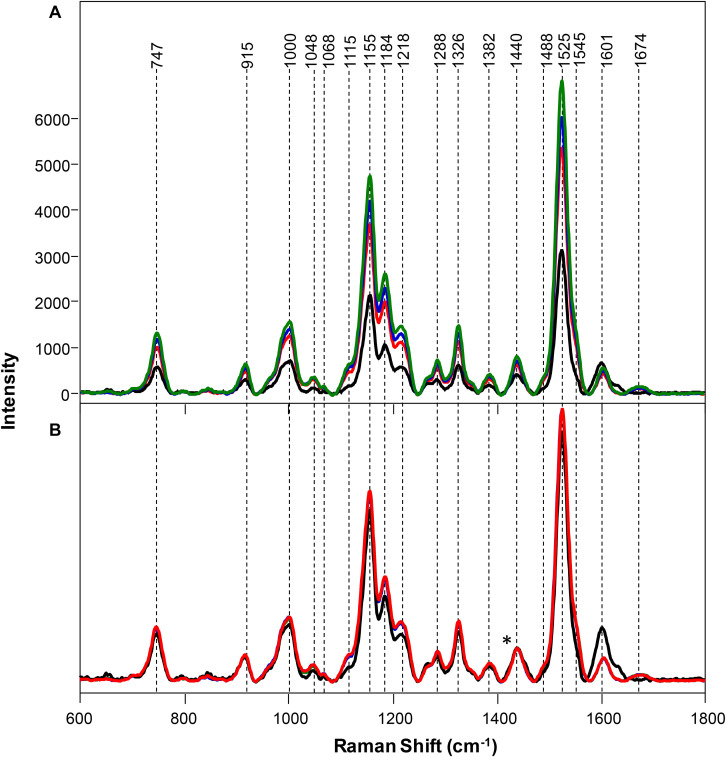
Raw **(A)** and normalized on 1382 cm^– 1^ band. **(B)** Raman spectra of healthy (green) rice and rice with N (black), P (blue), and K (red) deficiencies. Difference spectra are shown in the Supplementary Figure S2. The 1440 cm^−1^ peak, which was used for spectral normalization, is indicated by an asterisk (*).

Spectra collected from leaves of healthy rice plants exhibited vibrational bands that could be assigned to pectin (747 cm^–1^), cellulose (915, 1048, 1068, 1115, and 1155 cm^–1^), xylan (1184 cm^–1^), carotenoids (1000, 1525, and 1545 cm^–1^), phenylpropanoids (∼1600 cm^–1^), protein (1674 cm^–1^), and aliphatic vibrations (1218, 1288, 1326, 1382, 1440, and 1488 cm^–1^) (Figure 1 and Table 1). Spectra collected from ND, PD, and KD plants exhibited lower intensities of vibrational bands that originated from pectin, cellulose, xylan, aliphatic vibrations, and carotenoids, relative to the corresponding bands in the spectra of healthy rice (Figure 1). These changes suggest that N, P, and K deficiencies can be associated with a decrease in the pectin, cellulose, xylan, and carotenoid content in rice. Although we did not observe substantial changes in the intensity of amide I bands in the spectra collected from PD and KD rice, the intensity of this band was substantially lower in spectra collected from the leaves of ND rice. This finding suggests a decrease in the protein content of leaves associated with ND. This might be explained by one of the key roles of N in plants: N is the central element of all proteins, enzymes, and nucleic acids (Novoa and Loomis, 1981). Lastly, we found that spectra collected from ND plants exhibited an increase in the 1604 cm^–1^ band, which can be assigned to phenylpropanoids, whereas plants with PD and KD did not exhibit this spectral change. This suggests that ND in rice is associated with an increase in the phenylpropanoids content. We also found a small spectral shift of this band when comparing spectra collected from healthy (1605 cm^–1^) and ND (1602 cm^–1^) rice. This spectral shift indicates a change in the chemical composition of phenylpropanoids occurs in ND plants. It should be noted that this band shift was not evident in the spectra collected from PD and KD plants.

**TABLE 1:** Vibrational bands and their assignments for spectra collected from healthy ND, PD, and KD plants, as well as from rice with salt stress.

**Band**	**Vibrational mode**	**Assignment**
747	γ(C–O-H) of COOH	Pectin (Synytsya et al., 2003)
915	ν(C-O-C) in plane, symmetric	Cellulose, lignin (Edwards et al., 1997)
1000	ν_3_ (C-CH_3_ stretching) and phenylalanine	Carotenoids (Tschirner et al., 2009; Kurouski et al., 2015)
1048–1068	ν(C-O) + ν(C-C) + δ(C-O-H)	Cellulose (Almeida et al., 2010)
1115	COH bending	Cellulose (Almeida et al., 2010)
1155	asym ν(C-C) ring breathing	Cellulose (Edwards et al., 1997)
1184	ν(C-O-H) next to aromatic ring + σ(CH)	Xylan (Mary et al., 2012; Agarwal, 2014)
1218	δ(C-C-H)	Aliphatic (Yu et al., 2007), xylan (Agarwal, 2014)
1288	δ(C-C-H)	Aliphatic (Yu et al., 2007)
1326	δCH_2_ bending vibration	Cellulose, lignin (Edwards et al., 1997)
1382	δCH_2_ bending vibration	Aliphatic (Yu et al., 2007)
1440	δ(CH_2_) + δ(CH_3_)	Aliphatic (Yu et al., 2007)
1488	δ(CH_2_) + δ(CH_3_)	Aliphatic (Yu et al., 2007)
1527–1545	-C = C- (in plane)	Carotenoids (Adar, 2017; Devitt et al., 2018)
1601–1604	ν(C-C) aromatic ring + σ(CH)	Phenylpropanoids (Stewart et al., 2001; Agarwal, 2006; Jurasekova et al., 2006; Kang et al., 2016)
1674	C = O stretching, amide I	Proteins (Devitt et al., 2018)

Changes in the phenylpropanoid content of plants that were ND becomes even more prominent upon spectral normalization of the intensity of 1382 cm^–1^ band, which was assigned to CH_2_ vibration (Figure 1; Farber et al., 2019a). This chemical group is present in virtually all biological molecules in plants, making it unbiased by condition and effective for normalization. The normalized spectra of healthy, PD, and KD samples exhibited very similar profiles, with only small spectral changes. However, Raman spectra collected from ND exhibited a decrease in the intensities of vibrational bands that could be assigned to pectin, cellulose, xylan, aliphatic vibrations, and carotenoids as well as an increase in the intensity of phenylpropanoid vibration.

An increase in the intensity of phenylpropanoids revealed by RS in ND plants might be partially explained by an increase in the concentration of *p*-coumaryl and coniferyl alcohols, the precursors of H- and G-lignins. This assumption is based on the results of chromatographic analyses of rice plants made by Chishaki and Horiguchi (1997). These researchers observed more than a fourfold increase in *p*-coumaric and ferulic acids upon ND in rice leaves. However, carboxylic groups have distinct vibrational bands around 1700 cm^–1^ (Sanchez et al., 2020b), which were not observed in the collected Raman spectra of ND plants. Also, *p*-coumaric and ferulic acids contain a second vibrational band in this spectra region around 1630 cm^–1^, which was not observed in the spectra collected from ND plants. This experimental evidence suggests that an observed increase in the concentration of phenylpropanoids upon ND is unlikely to be associated with an increase in the concentration of *p*-coumaric and ferulic acids. HPLC-MS analysis of biochemical changes in rice upon ND reported by Steward and co-workers suggests that an increase in the concentration of phenylpropanoids can be due to an increased concentration of kaempherol, quercetin and its derivative isorhamnetin (Stewart et al., 2001). Spectroscopic analysis of these compounds reported by Jurasekova and co-authors indicate that quercetin’s phenolic vibrational band was at 1610 cm^–1^, whereas kaempherol’s phenolic vibrational band was at 1604 cm^–1^ (Jurasekova et al., 2006). It should be noted that we observed a blue shift of the phenolic band upon the development of N deficiency by plants (Supplementary Figure S3). Based on this experimental evidence, we can conclude that the observed increase in phenolic band is likely to be assigned to the increased concentration of kaempherol in rice leaves.

A decrease in the intensity of carotenoid vibrations suggests a decrease in the concentration of carotenoids upon ND. RNA sequencing of ND rice seedling roots indicated activation of the *PSY3* gene, which regulates abscisic acid (ABA) biosynthesis (Hsieh et al., 2018). ABA is synthesized from carotenoids and functions as a plant signaling molecule upon various abiotic stresses (Li et al., 2008; Welsch et al., 2008). Thus, a decrease in the carotenoids upon ND could be partially attributed by their conversion into signaling molecules that are synthesized by plants as a stress response.

Next, we used PLS-DA to determine whether RS can be used for the quantitative identification of these nutrient deficiencies based on the spectroscopic signatures of rice leaves. We also evaluated how early RS can predict the appearance of such deficiencies.

Our results demonstrated that ND, PD, and KD could be predicted as early as D2, with 84.6% on average. The most accurate predictions were made for ND (93.9%), whereas the least accurate predictions were made for KD (79.6%). The average accuracy of diagnostics increased at D4 (96.9%), thereafter remaining above 90% (90.7% at D6, 98.1% at D8, and 93.5% at D11) (Table 2).

**TABLE 2:** Total average of binary models for N, P, and K stresses.

	**D2 (%)**	**D4 (%)**	**D6 (%)**	**D8 (%)**	**D11 (%)**
ND	93.9	98.7	100.0	100.0	100.0
PD	80.4	95.3	86.3	94.3	90.4
KD	79.6	96.7	86.0	100.0	90.0

**FIGURE 2:**
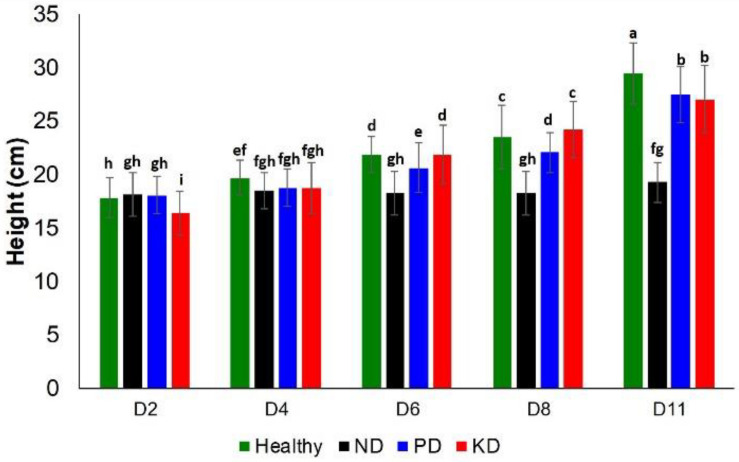
Histogram of a change in the plant height at D2, D4, D6, D8, and D8 for healthy (green), ND, PD, and KD rice plants. Each bar represents the mean ± SE (*n* = 30). Different letters in each graph **(a–i)** indicate significant differences (*P* < 0.05, ANOVA and Duncan test).

Next, we evaluated the impact of these nutrient changes on plant height and chlorophyll content at D2, D4, D6, D8, and D11. With regard to plant height, ND and PD could be differentiated among other groups at D6 and D8. However, at D11, the only notable difference in plant height was for ND, which was the shortest group (Figure 2). Thus, plant height is a relatively poor approach to detect nutrient deficiencies in rice plants.

**FIGURE 3:**
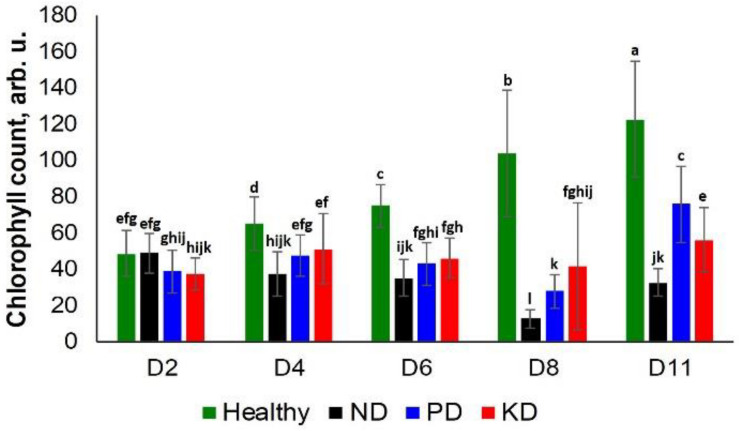
Histogram of a change in the chlorophyll content of plants at D2, D4, D6, D8, and D8 for healthy (green), ND, PD, and KD rice plants. Each bar represents the mean ± SE (*n* = 30). Different letters in each graph **(a–l)** indicate significant differences (*P* < 0.05, ANOVA and Duncan test).

We observed a significant difference in the chlorophyll content of ND plants and other groups at D4. However, only a slightly different decrease was observed in the chlorophyll content of the PD and KD groups at the same time point (Figure 3). Similarly, at D6, all test groups exhibited a decrease in chlorophyll content relative to the control group rice. However, this decrease was not specific to a particular nutrient deficiency until D8. At this time point, ND plants had the lowest chlorophyll counts, followed by PD, KD, and the control plants. At D11, the trend was slightly different: ND had the lowest chlorophyll counts, followed by KD, PD, and the control plants (Figure 3). Thus, we suggest that chlorophyll density can be used to differentiate ND from PD and KD with high confidence only at D8. Carotenoids also have multiple roles in photosynthesis, including photochemical and non-photochemical processes (Frank and Cogdell, 1996). The drastic decline in chlorophyll content of the ND rice plants may partially be attributed to the decrease of carotenoid concentration (Figure 1 and Table 1).

Chlorotic symptoms could be detected by visual examination of plants at D6. Precise visual analysis of plants also enabled the detection of dry tips of leaves that appeared on PD and KD plants at D11 (Supplementary Figure S4). However, RS demonstrated an average of 84.6% accuracy in predicting ND, PD, KD nutrient deficiency earlier, at D2. This accuracy increased to 93.9% at D4 and remained above 90% thereafter.

### Raman-Based Diagnostics of Salt Stress

**FIGURE 4:**
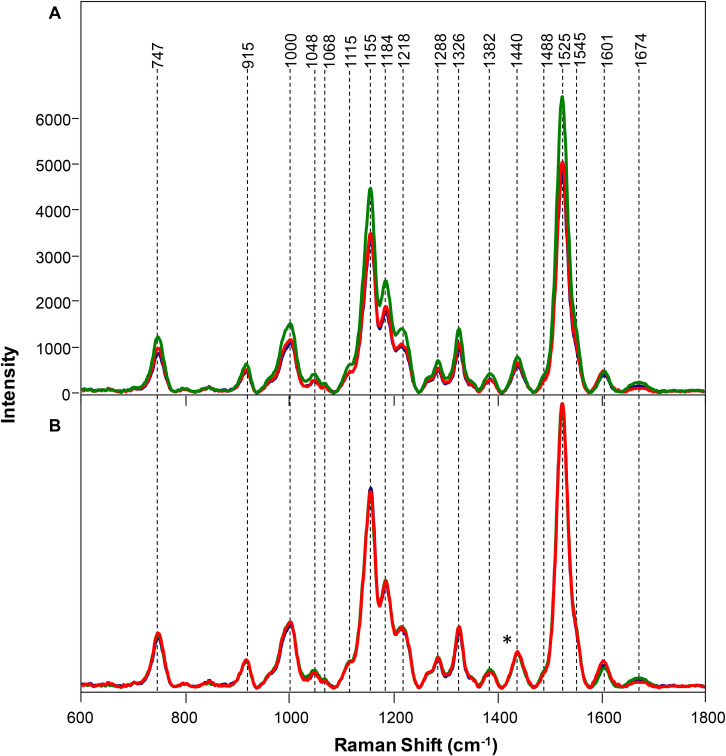
Raw **(A)** and normalized on 1382 cm^– 1^ band. **(B)** Raman spectra of healthy (green), rice and rice with 80 mM (red), and 120 mM (blue) salinity stresses. Difference spectra are shown in the Supplementary Figure S5. The 1440 cm^−1^ peak, which was used for spectral normalization, is indicated by an asterisk (*).

Symptomatic plants with medium and high salinity stresses exhibited decreased intensity of vibrational bands that could be assigned to pectin (747 cm^–1^), cellulose (915, 1048, 1068, 1115, and 1155 cm^–1^), xylan (1184 cm^–1^), carotenoids (1000, 1525, and 1545 cm^–1^), phenylpropanoids (∼1600 cm^–1^), protein (1674 cm^–1^), and aliphatic vibrations (1218, 1288, 1326, 1382, 1440, and 1488 cm^–1^) (Figure 4).

At the same time, normalized spectra did not reveal substantial spectral changes (Figure 4). This suggests that salt stress causes very small transformations in the scaffold molecules of rice. We used PLSD-DA to determine whether such small changes could be used for confirmatory diagnostics of medium and high salinity stresses. Our results showed that as early as D2, RS could determine salinity stress with an average accurate identification of 91.7% (Table 3). As expected, high salinity stress caused more substantial changes in the plant, which was reflected by higher prediction accuracy (94.5%) relative to medium salinity (89.0%) stress. The average prediction accuracy of both medium and high salinity stresses at D4 was 82.5%; at D6, the accuracy was 96.0% for the medium salinity stress. It should be noted that plants exposed to high salinity were found nearly scorched by D6; therefore, no Raman measurements were taken from their leaves.

**TABLE 3:** Total average of binary models for medium and high salinity stresses.

	**D2 (%)**	**D4 (%)**	**D6 (%)**
Medium (80 mM) salinity stress	89.0	81.7	96.0
High (120 mM) salinity stress	94.5	83.3	–

**FIGURE 5:**
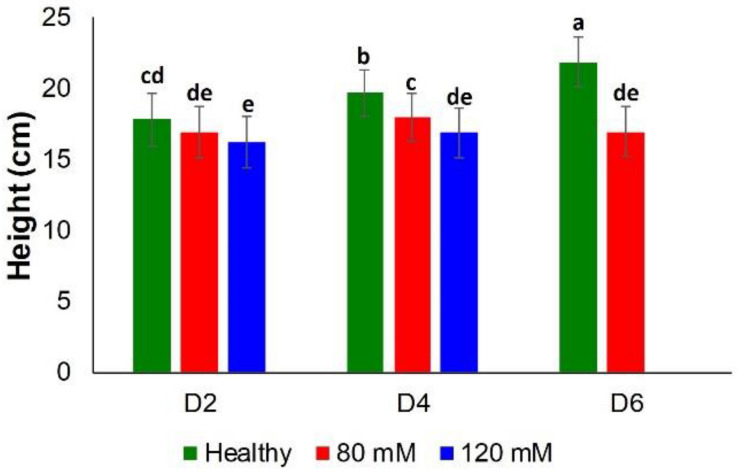
Histogram of a change in the plant height at D2, D4, D6, D8, and D8 for healthy (green), 80 and 120 mM salinity stresses. Each bar represents the mean ± SE (*n* = 30). Different letters in each graph **(a–e)** indicate significant differences (*P* < 0.05, ANOVA and Duncan test).

Classical approaches to elucidate salinity stresses (height measurements and chlorophyll content) were not sufficiently accurate, especially at earlier time points. Specifically, there was no substantial difference between the heights of plants in all three groups, until D4 (Figure 5).

**FIGURE 6:**
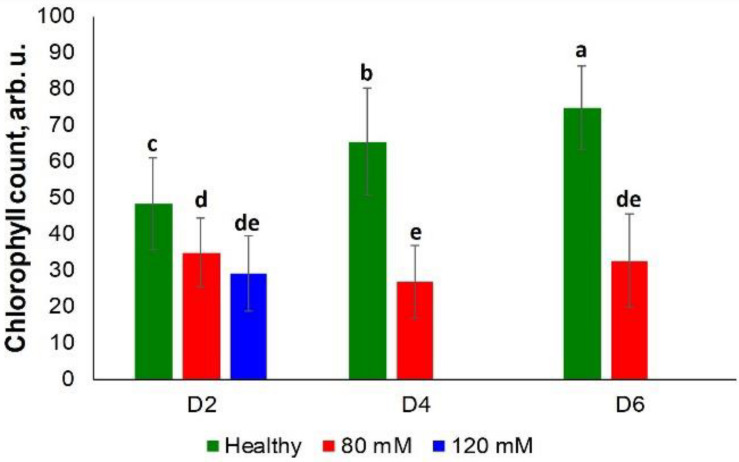
Histogram of a change in the chlorophyll content of plants at D2, D4, D6, D8, and D8 for healthy (green), 80 and 120 mM salinity stresses. Each bar represents the mean ± SE (*n* = 30). Different letters in each graph **(a–e)** indicate significant differences (*P* < 0.05, ANOVA and Duncan test).

Rice exposed to both medium and high salinity stresses did not show substantial differences in the chlorophyll content at D2 (Figure 6). However, differences were observed at D4 and D6 among the control plants and those plants experiencing medium salinity stress. However, plants were in poor condition due to high salinity stress at these time points (D4 and D6), which prevented determination of the chlorophyll content from this group of plants (Supplementary Figure S6).

These results suggest that the combination of RS and chemometrics can be used for highly accurate diagnostics of salinity stresses on plants.

## Conclusion

This study demonstrated the power of RS for label-free, non-invasive, and non-destructive detection and identification of ND, PD, and KD, as well as salinity stresses on rice plants. Our results showed that in early stages (D2), these stresses could be predicted with high accuracy and identified with only 1 s of spectral acquisition. Our results also demonstrated that RS could be used to reveal changes in the scaffold molecules of plants that are associated with nutrient deficiencies and salinity stresses. These spectroscopic changes can facilitate the elucidation of the molecular mechanisms of plant responses to various biotic and abiotic stresses. Considering the high sensitivity of RS for the diagnostics of biotic stresses on plants (Egging et al., 2018; Farber and Kurouski, 2018; Farber et al., 2019a,b; Sanchez et al., 2019a,b, 2020a), one can expect that this spectroscopic approach has far-reaching implications in various disciplines, from basic plant biology and pathology to agriculture and horticulture.

## Data Availability Statement

The raw data supporting the conclusions of this article will be made available by the authors, without undue reservation.

## Author Contributions

LS: investigation, data curation, and methodology. AE: data curation. SB: methodology. ES: methodology and supervision. DK: methodology, funding acquisition, and supervision. All authors contributed to the article and approved the submitted version.

## Conflict of Interest

The authors declare that the research was conducted in the absence of any commercial or financial relationships that could be construed as a potential conflict of interest. The reviewer, NA, declared a shared affiliation, with no collaboration, with several of the authors, LS, AE, SB, ES, and DK, to the handling editor at the time of review.
