# Supplementary material for: Raman Spectroscopy Enables Non-invasive and Confirmatory Diagnostics of Salinity Stresses, Nitrogen, Phosphorus, and Potassium Deficiencies in Rice
Source: Front Plant Sci. 2020 Oct 22;11:573321. doi: 10.3389/fpls.2020.573321 (PMC7642205; doi:10.3389/fpls.2020.573321)
Supplement: Supplementary file 1 [file Data_Sheet_1.PDF]

Supporting Information

Raman Spectroscopy Enables Non-Invasive and Confirmatory Diagnostics of Salinity Stresses, Nitrogen, Phosphorus, and Potassium Deficiencies in Rice

Lee Sanchez[1], Alexei Ermolenkov[1], Sudip Biswas[2], Endang M. Septiningsih[2], and Dmitry Kurouski[*1,3]

1. Department of Biochemistry and Biophysics, Texas A&M University, College Station, Texas 77843, United States
2. Department of Soil and Crop Sciences, Texas A&M University, College Station, Texas 77843, United States
3. The Institute for Quantum Science and Engineering, Texas A&M University, College Station, Texas, 77843, United States

Constitution of stock Youshida solution:

Control: NH_4NO_3 114.30 mg/L, $NaH_2PO_4.2H_2O$ 50.40 mg/L, K_2SO_4 89.30 mg/L, $CaCl_2$ 108.25 mg/L and $MgSO_4.7H_20$ 405 mg/L.

Nitrogen deficient [ND]: NH_4NO_3 0 mg/L, $NaH_2PO_4.2H_2O$ 50.40 mg/L, K_2SO_4 89.30 mg/L, $CaCl_2$ 108.25 mg/L and $MgSO_4.7H_20$ 405 mg/L.

Phosphorus deficient [PD]: NH_4NO_3 114.30 mg/L, $NaH_2PO_4.2H_2O$ 0 mg/L, K_2SO_4 89.30 mg/L, $CaCl_2$ 108.25 mg/L and $MgSO_4.7H_20$ 405 mg/L.

Potassium deficient [KD]: NH_4NO_3 114.30 mg/L, $NaH_2PO_4.2H_2O$ 50.40 mg/L, K_2SO_4 0 mg/L, $CaCl_2$ 108.25 mg/L and $MgSO_4.7H_20$ 405 mg/L.

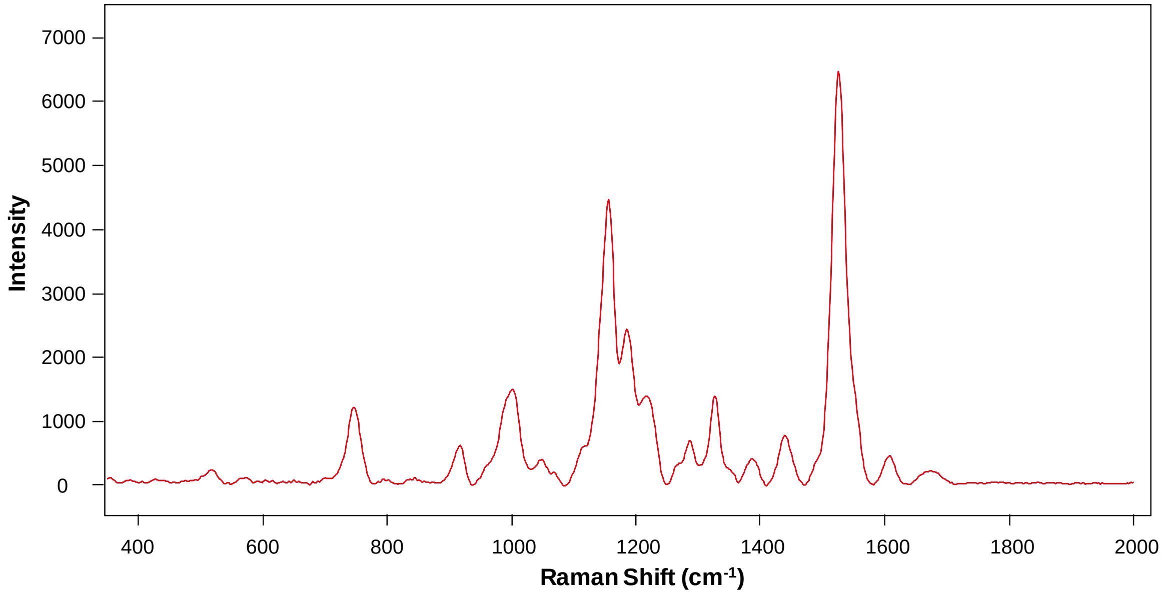

Figure S1. Raman spectra collected from the same plants in the control group of rice at D2 (red) and D11 (blue).

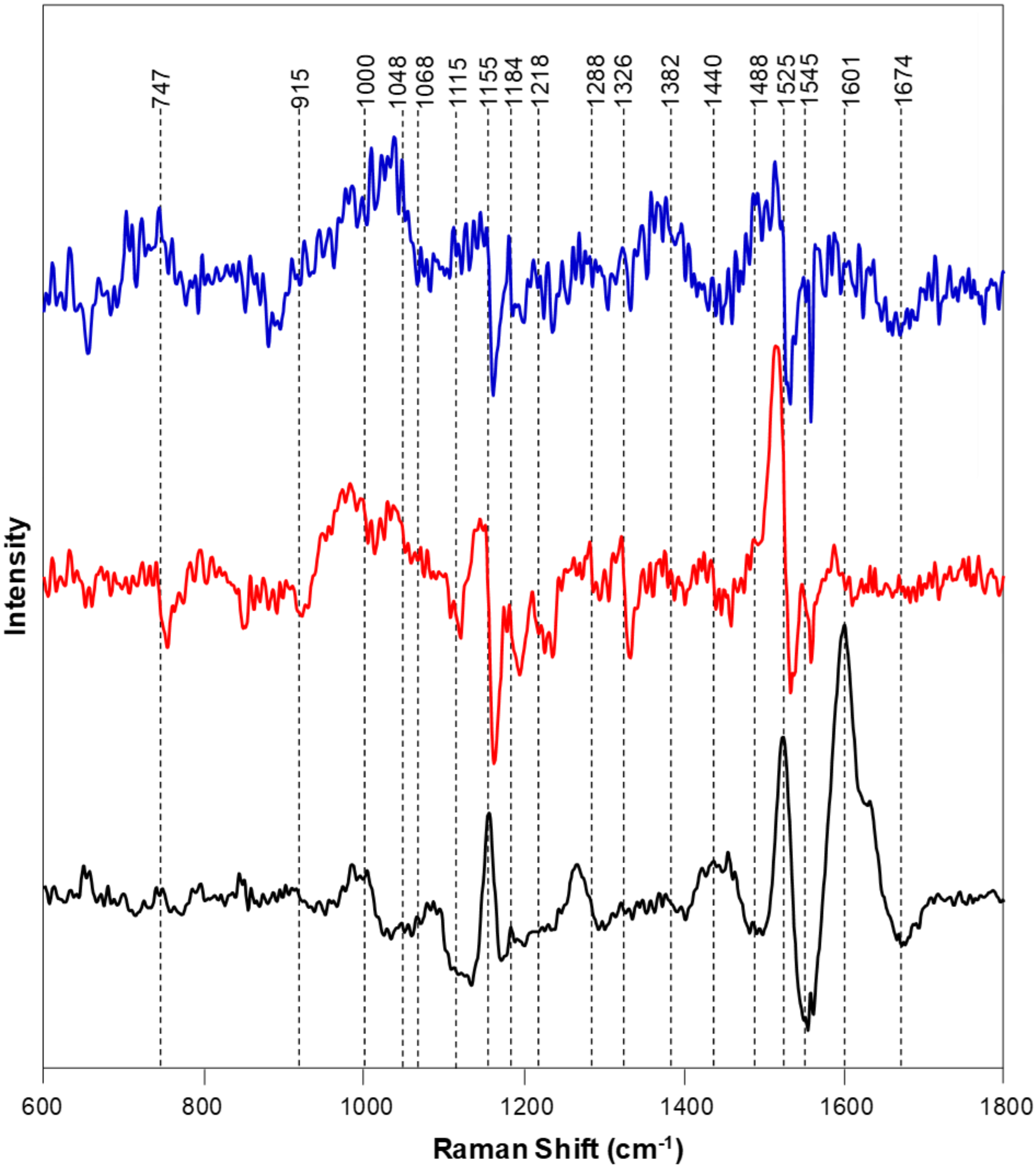

Figure S2. Difference Raman spectra of rice with N (black), P (blue) and K (red) deficiencies.

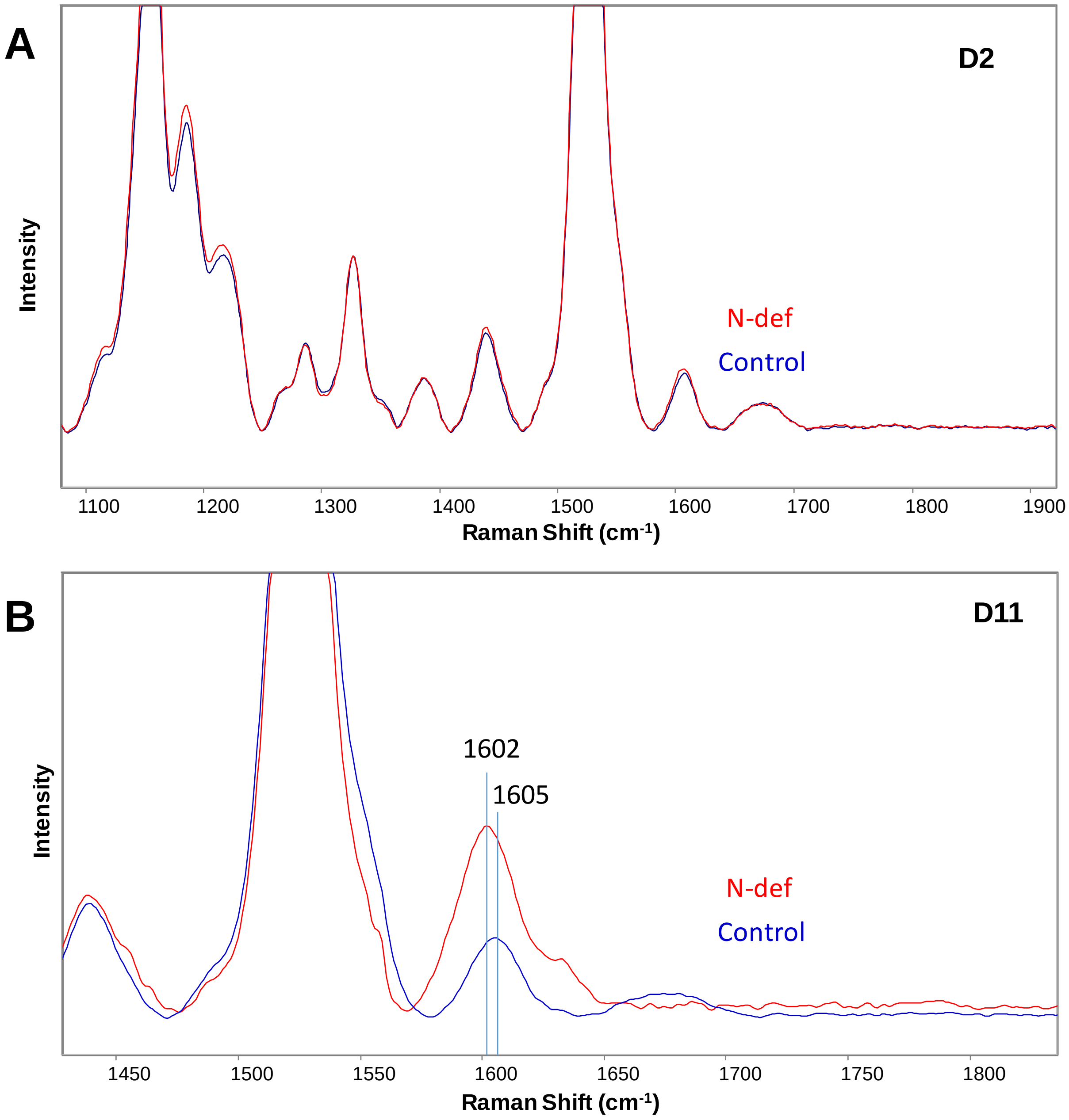

Figure S3. Raman spectra collected from control and ND plants at D2 (A) and D11 (B).

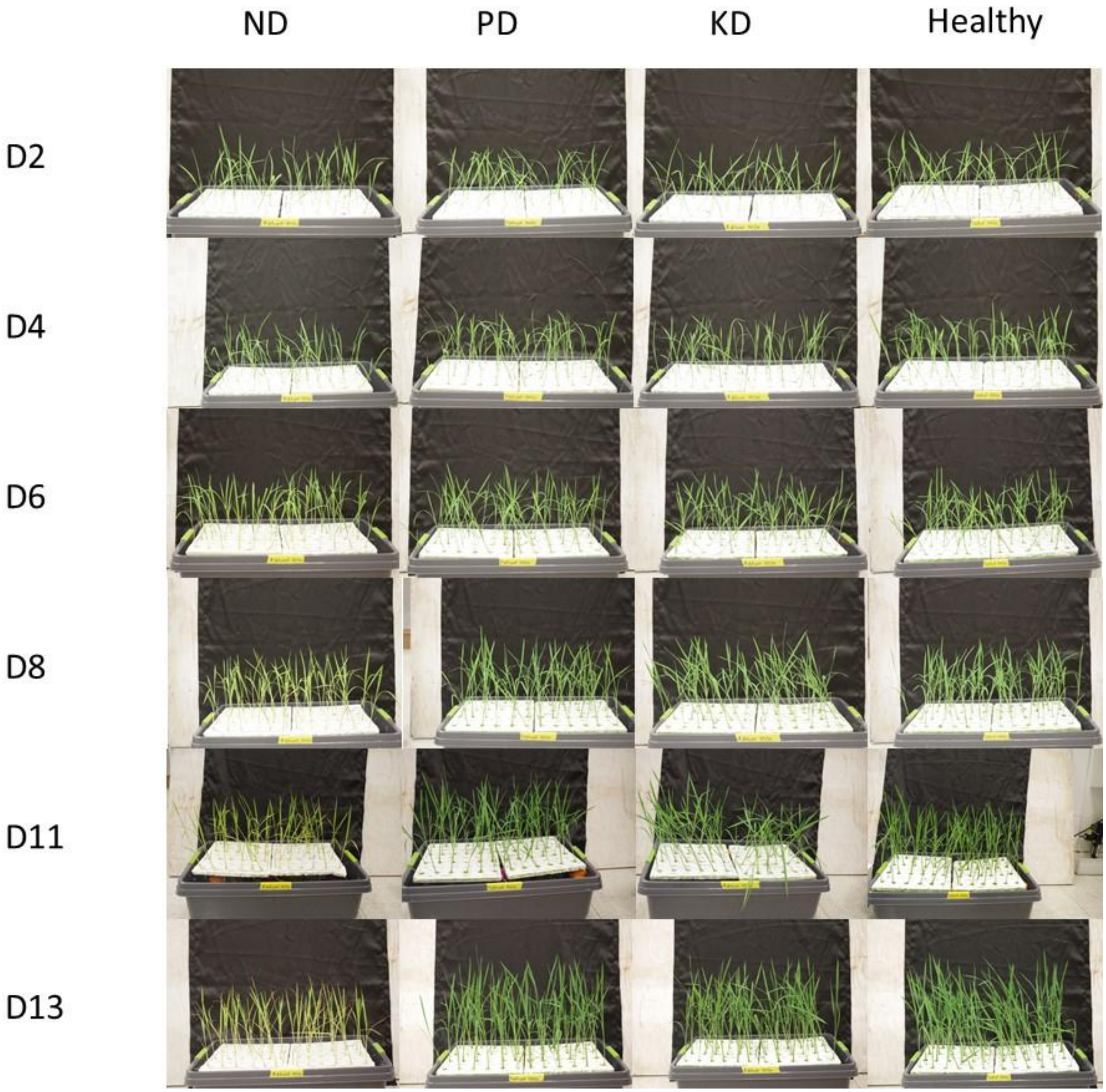

Figure S4. Photographs of healthy plants and plants exposed to ND, PD and KD at D2, D4, D6, D8, D11 and D13.

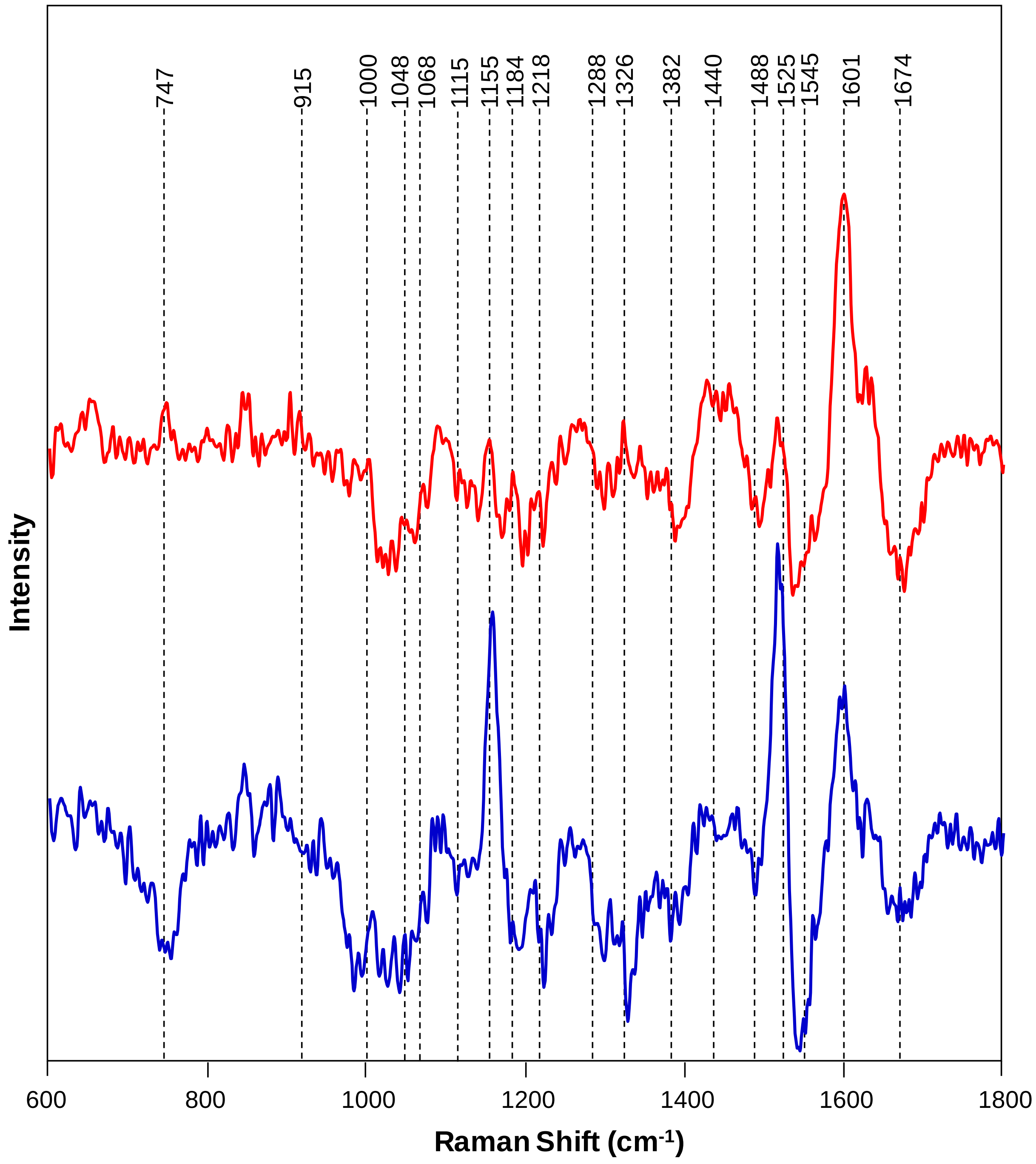

Figure S5. Difference Raman spectra of rice with 80 mM (red) and 120 mM (blue) salinity stresses.

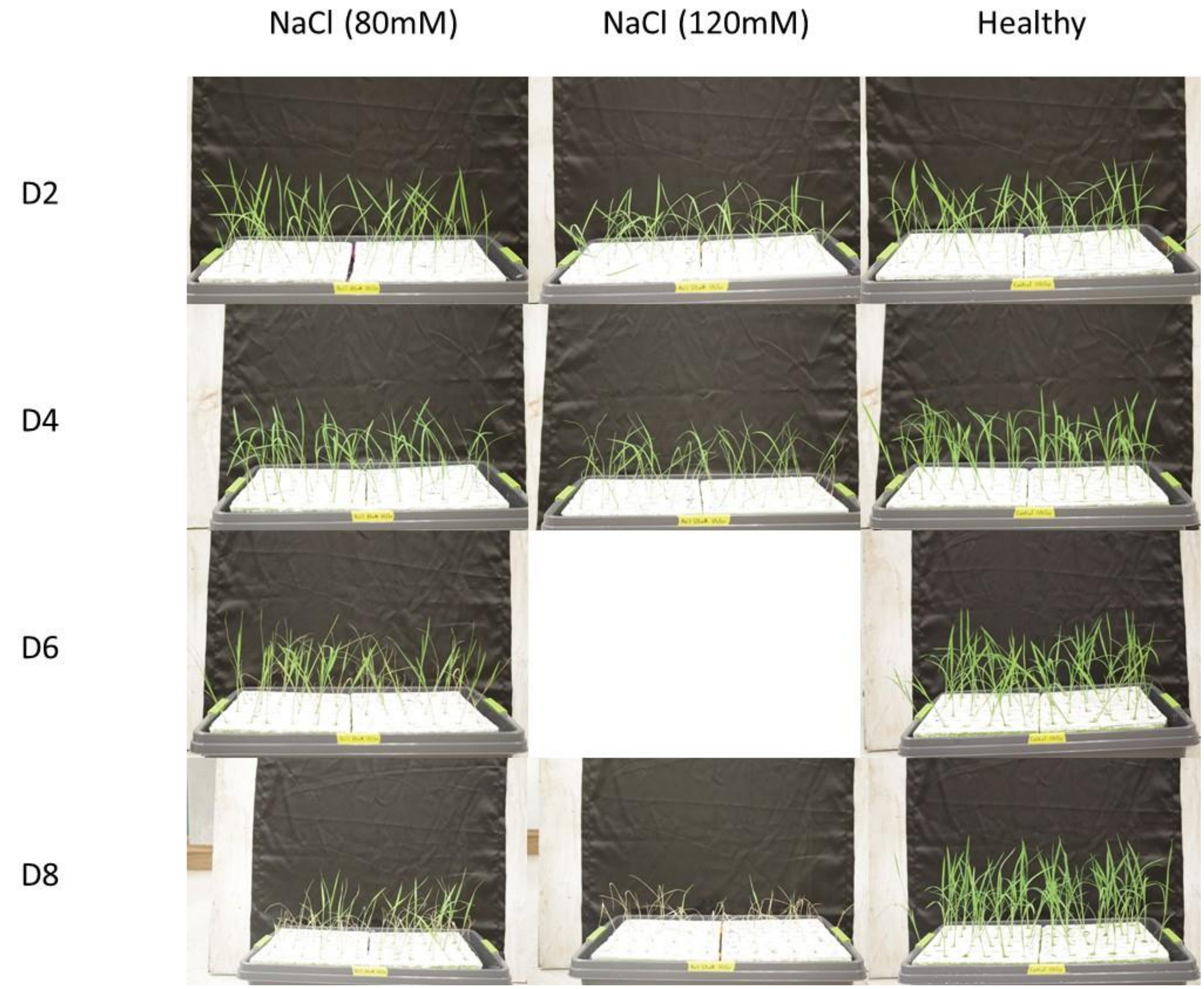

Figure S6. Photographs of healthy plants and plants exposed to medium (80 mM) and high (120 mM) salinity stresses at D2, D4, D6 and D8.
